# Supplementary material for: Atraric Acid Exhibits Anti-Inflammatory Effect in Lipopolysaccharide-Stimulated RAW264.7 Cells and Mouse Models
Source: Int J Mol Sci. 2020 Sep 25;21(19):7070. doi: 10.3390/ijms21197070 (PMC7582958; doi:10.3390/ijms21197070)
Supplement: Supplementary file 1 [file ijms-21-07070-s001.pdf]

International Journal of
Molecular Sciences

MDPI

Supplementary

Atraric Acid Exhibits Anti-Inflammatory Effect in Lipopolysaccharide-Stimulated RAW264.7 Cells and Mouse Models

Seul-Ki Mun [1,†], Kyung-Yun Kang [2,†], Ho-Yeol Jang [2], Yun-Ho Hwang [1], Seong-Gyeol Hong [1], Su-Jin Kim [1], Hyun-Wook Cho [3], Dong-Jo Chang[1], Jae-Seoun Hur [4] and Sung-Tae Yee [1,*]

[1] Department of Pharmacy, Sunchon National University, 255 Jungang-Ro, Suncheon 549-742, Korea; motomoto1210@naver.com (S.-K.M.); hyh7733@naver.com (Y.-H.H.); hong9217@naver.com (S.-G.H.); ksz1353@naver.com (S.-J.K.), djchang@scnu.ac.kr (D.-J.C.)
[2] Suncheon Research Center for National Medicines, Suncheon 549-742, Korea; kang8404@nate.com (K.-Y.K.); yeol2686@naver.com (H.-Y.J.)
[3] Department of Biology, Sunchon National University, Suncheon 549-742, Korea; hwcho@sunchon.ac.kr
[4] Department of Environmental Education, Korea Lichen Research Institute, Sunchon National University, Suncheon 549-742, Korea; jshur1@sunchon.ac.kr
* Correspondence: sungtae@sunchon.ac.kr; Tel.: +82 61 750 3752; Fax: +82 61 750 3708
† These authors contributed equally to this work.

Received: 26 August 2020; Accepted: 22 September 2020; Published: date

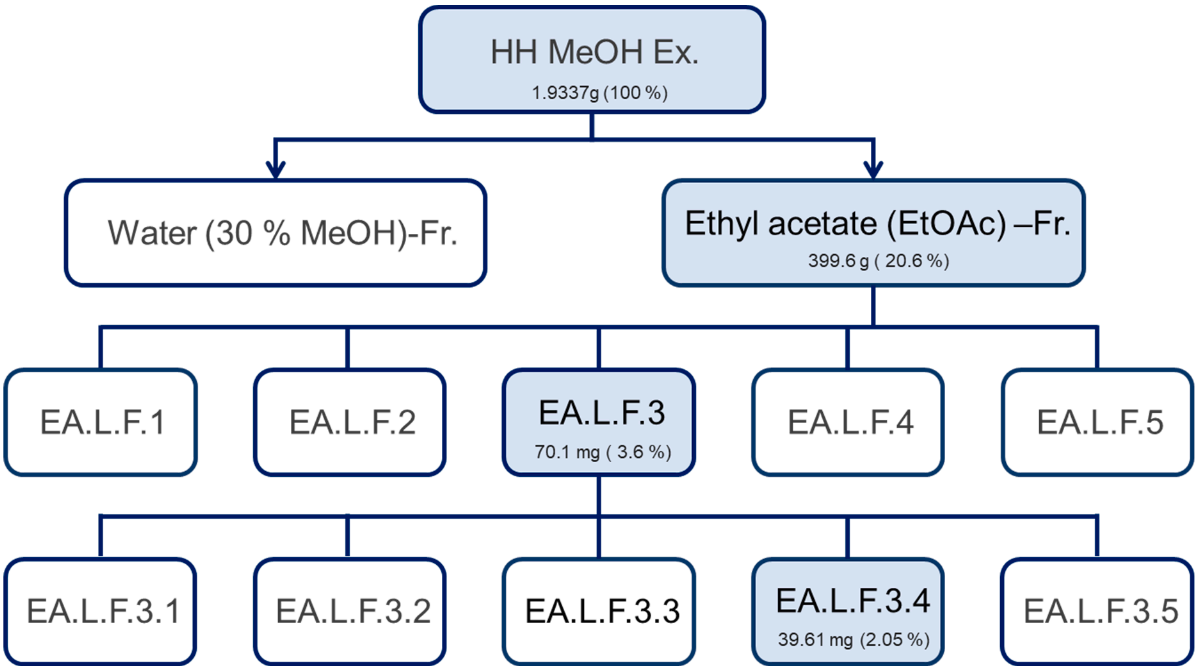

Figure 1. Procedure for fractionation and separation by HH MeOH extracts (Ext.). Final fraction is atraic acid (EA.L.F.3.4). Abbreviations: HH, *Heterodarmia hypoluca*; EA. L.F, Ethyl acetate layer fraction.

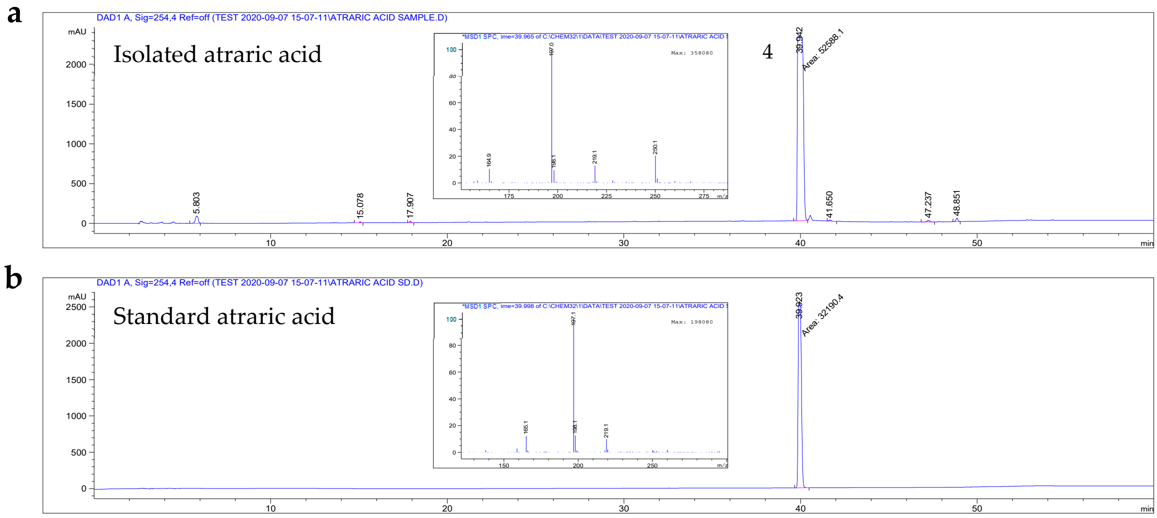

Figure 2. HPLC chromatograms profiles of atraric acid. LC-MS/ELSD analysis was performed to analyze the purified atraric acid. As analysis conditions, the initial solvent 5% solvent B hold at for 5min. The gradient condition, Solvent B was than increased from 5% to 70% within 40min, following proportions solvent B; 45-50min, 70-100%, hold at B; 100% for 10min. (**a**) atraric acid iolated from HH, (**b**) standard.

Table 1. Integration of chromatogram.

#	Time	Area	Height	Width	Area%
1	5.803	1041.4	90.5	0.1583	1.913
2	15.078	73.9	11.4	0.0975	0.136
3	17.907	98.1	15.7	0.0968	0.18
4	39.942	52588.1	2316.2	0.3784	96.598
5	41.65	112.2	13.3	0.1281	0.206
6	47.237	194.2	19.9	0.1516	0.357
7	48.851	332.3	42.7	0.1208	0.61
